# CPP-calcification of articular cartilage is associated with elevated cytokine levels in synovial fluid

**DOI:** 10.3389/fcell.2025.1535530

**Published:** 2025-03-19

**Authors:** Sina Stücker, Franziska Koßlowksi, Adrian Buchholz, Andrea Schwab, Agnieszka Halm-Pozniak, Christoph H. Lohmann, Jessica Bertrand

**Affiliations:** Department of Orthopaedic Surgery, Otto-von-Guericke-University, Magdeburg, Germany

**Keywords:** calcification, cartilage, synovial fluid, senescence, calcium crystals

## Abstract

**Background:**

Calcification of articular tissues is commonly observed in later osteoarthritis (OA) stages and can be caused by basic calcium phosphate (BCP) or calcium pyrophosphate (CPP) crystals. Calcification, particularly CPP deposition, has recently been associated with inflammation and cellular senescence. Investigating this association, we analyzed the concentration of various inflammatory mediators in synovial fluid and synovial membrane of OA patients in relation to calcification and the different crystal types.

**Methods:**

Synovial fluid was collected from OA patients during joint replacement surgery. Cytokine concentrations were measured using magnetic bead-based multiplex assay using Luminex® technology. Radiographs were used to determine and grade calcification of the knee joint and involved calcium crystal types were identified via Raman spectroscopy.

**Results:**

Synovial fluid of patients with radiological calcification showed elevated levels of multiple cytokines (IL-10, IL-15, IL-1ra, GM-CSF), chemokines (IL-8, MCP-1, MIP-1b) and growth factors (PDGF-AB/BB, VEGF). Crystal differentiation revealed higher synovial fluid concentrations of IL-15, IL-1ra, IL-10, GM-CSF, PDGF-AB/BB and MIP-1b in patients with CPP- compared to BCP-calcified cartilage.

**Conclusion:**

We show an elevated cytokine profile in synovial fluid of patients with radiological calcification that may be linked to CPP depositison in cartilage.

## 1 Introduction

Calcification of articular soft tissue is commonly observed in degenerative crystallopathies such as osteoarthritis and is caused by the accumulation of calcium crystals in the extracellular space. Calcium crystals can deposit in all articular soft tissues, including hyaline cartilage, ligaments, menisci and synovium. By shedding from these deposits, some crystal types can frequently be found in synovial fluid aspirates under polarized light microscopy (Cheung and McCarty, 1985). These calcium-containing crystals can be divided into two types: basic calcium phosphate (BCP) crystals and calcium pyrophosphate dihydrate (CPP) crystals. BCP is the most common crystal type in OA joints) and shows a strong correlation with radiographic and histological disease severity (Fuerst et al., 2009; Nalbant et al., 2003). On cellular level, BCP crystals are closely linked to chondrocyte hypertrophy and canonical Wnt signalling (Bertrand et al., 2018). Instead, CPP crystals cause CPP deposition disease (CPPD). CPPD describes a heterogeneous group of clinical manifestations including acute and chronic gout-like flares and often manifests as radiologically visible linear or punctuate opacities within the joint space (Cowley and McCarthy, 2023). The incidence of CPPD increases with age and CPP crystals have been linked to the cellular phenotype of senescence (Felson et al., 1989; Mitsuyama et al., 2007). The details of this association remain elusive, though.

Cellular senescence describes a state of irreversible permanent cell cycle arrest in the G1 phase to restrict the proliferation of damaged cells. Various stressors are known to induce senescence, including excessive mechanical loading, telomere shortening, mitochondrial dysfunction, oxidative stress and DNA damage (Coryell et al., 2021). Senescent cells can display a characteristic senescence-associated secretory phenotype (SASP), which entails the production of various non-exclusive factors including pro-inflammatory cytokines, chemokines, proteases and growth factors, creating a pro-inflammatory microenvironment and reinforcing cell cycle arrest (Coryell et al., 2021). Pro-inflammatory cytokines such as interleukins IL-1 and IL-15 or Granulocyte-macrophage colony-stimulating factor (GM-CSF) represent the most commonly reported SASP factors (Yue et al., 2022). Senescence can propagate to neighbouring cells in paracrine manner via SASP (Acosta et al., 2013).

Senescent chondrocytes were shown to accumulate in osteoarthritic cartilage, specifically in damaged areas and near lesions (Price et al., 2002), and promote cartilage destruction via the production of catabolic matrix-degrading enzymes such as matrix metalloproteinases (Forsyth et al., 2005). In fact, the degree of chondrocyte senescence correlates with cartilage damage and disease severity (Price et al., 2002), participating in OA development and progression of OA (Georget et al., 2023). Chondrocyte senescence has also been linked to cartilage calcification (Richter et al., 2023; Meyer et al., 2021).

Similarly, senescent fibroblast-like synoviocytes may also contribute to OA progression (Xiang et al., 2022). During OA progression, the synovium is infiltrated by immune cells, displaying hyperplasia and calcification (Jeon et al., 2018). However, it remains unclear whether these synovial changes are primary or merely an epiphenomenon of joint inflammation or cartilage degradation.

In respect to the association between senescence and OA, targeting senescence may therefore be a promising therapeutic strategy for OA. Pre-clinical studies demonstrate the potential of selective transgenic or pharmacological removal of senescent cells in attenuating pain behaviour and cartilage degeneration in a mouse models of post-traumatic (Jeon et al., 2017) and spontaneous OA (Gil et al., 2022). Senolytic treatment also reduced vascular calcification and osteogenic signalling in aged mice (Roos et al., 2016). However, the link between articular calcification and cellular senescence remains elusive. Therefore, this study aims to investigate the association between senescence and articular calcification by measuring the expression of various inflammatory mediators in synovial fluid and synovial membrane of OA patients with regards to the presence and type of articular calcification.

## 2 Materials and methods

### 2.1 Sample collection and preparation

Human synovial fluid, synovial membrane and tibia cartilage samples were obtained from OA patients undergoing knee joint replacement. All donors provided written informed consent before surgery. All procedures were approved by the Institutional Review Board (IRB) of the Medical School, Otto-von-Guericke University Magdeburg (IRB No. 28/20). The Kellgren Lawrence-Score was determined based on radiograph evaluation (Kellgren and Lawrence, 1957). Presence and severity of radiological calcification was scored according to a grading system from 0 (no visible calcification) to 3 (severe calcification in the joint space) as described before (Stücker et al., 2024) (Supplementary Figure S1A). Cartilage, synovial membrane and synovial fluid samples were snap frozen in liquid nitrogen and stored at −80° until further processing.

### 2.2 Multiplex cytokine assay

To measure the concentration of various senescence-associated mediators in synovial fluid and synovial membrane, the Human XL Cytokine Luminex® Performance Assay 44-plex Fixed Panel (LKTM014, R&D Systems Inc. Minneapolis, United States) was performed according to the manufacturer’s instructions. Synovial fluid samples were thawed, centrifuged at 16000G for 4 min. Synovial membrane samples were thawed and 10 mg of tissue was homogenized in 1 mL PBS with protease inhibitors (cOmplete mini EDTA free, Roche, Basel, Switzerland) using Precellys tubes (Tissue homogenizing CKMix, Bertin Technologies, France). All samples were diluted in supplied calibrator diluent RD6-65 prior to assaying. Mean fluorescence intensity was detected with a Bio-Plex analyzer (Bio-Rad Laboratories, Hercules, CA, United States) with a flow rate set to 60 μL/min, a reporter gain setting of 3,631 and a beat count of 50 count/region. Protein concentrations in synovial fluid and homogenized synovial membrane samples were quantified using the Pierce BCA protein assay kit (Thermo Scientific, Waltham, United States) to normalize cytokine concentrations.

### 2.3 Histology

Cartilage and synovial membrane samples were fixed in 4% paraformaldehyde (Fischar) for 24 h and dehydrated in ethanol, followed by paraffin embedding. Samples were cut into sections of 4 µm thickness on a microtome (Hyrax M55, Zeiss, Oberkochen, Germany). Von Kossa staining was performed to visualize calcifications and guide crystal identification via Raman spectroscopy. Hematoxylin eosin staining was performed to assess synovial inflammation according to the Krenn Score (Krenn et al., 2006).

### 2.4 Immunofluorescence staining

Cartilage and synovium sections were deparaffinised in xylene. Epitopes were retrieved using citrate pH 6.0 at 94°C for 25 min and blocked in 5% BSA. A primary antibody against p16 (1:500, proteinTech 10883-1-AP) was incubated overnight at 4°C. Control IgG staining was included to ensure antibody specificity. Alexa Fluor 555 (Thermo Scientific) was used as a secondary antibody and was incubated for 1 h at room temperature. Stained sections were mounted with Roti-Mount FluorCare DAPI (Carl Roth) and stored at 4°C until imaging. Stained sections were imaged on a Zeiss Axiocam 702 mono with a 10x/0.45 objective. Images were analysed using Fiji ImageJ (Version 1.54). After applying a threshold the stained area was quantified and normalized to the number of cells counted in the same image. Control IgG staining was substracted.

### 2.5 RNA extraction and quantitative RT-PCR

For isolation of total RNA, frozen cartilage samples were pulverized in liquid nitrogen and lysed in QIAZOL (QIAGEN). Chloroform was used for phase separation and RNA extraction was performed using the RNeasy® Plus Micro Kit (QIAGEN). Synovial membrane samples were homogenized in QUIAZOL in a Precellys tissue homogenizer (Bertin). RNA was extracted using chloroform, isopropanol and ethanol. Extracted RNA was diluted in nuclease-free water (Carl Roth) and the concentration was measured on a spectrophotometer (Infinite F200 Pro, Tecan). Extracted RNA was reverse transcribed into cDNA using the High-Capacity cDNA Reverse Transcription Kit (Thermo Scientific). Quantitative RT-PCR was performed with Sybr Green Mastermix (Thermo Scientific) on a QuantStudio 6. Primer sequences that were used are listed in the (Supplementary Table S2). Relative standard curves were included to quantify gene expression. Glyceraldehyde-3-phosphate dehydrogenase (GAPDH) was used as a housekeeping gene to normalize gene expression.

### 2.6 Raman spectroscopy

Crystal types in cartilage and synovium were identified by Raman spectroscopy as described previously (Stücker et al., 2024) using a confocal Raman microscope (Bruker Senterra II, OPUS Software 7.8). For sample preparation, paraffin-embedded tissue sections were deparaffinized with xylene and air dried. An overview image was taken using a 10x/0.25 objective to select regions of interest for measurement based on von Kossa staining of corresponding sections. Raman spectra were mapped with a 785 nm laser of 50 mW and a 10x/0.25 objective on a grid of measurement points with 10 µm distance. Measurement parameters were set to a spectral range of 50-4105 cm^−1^, covering the spectral range of crystal-specific signals at 960 cm^−1^ for BCP and 1050 cm^−1^ for CPP. Spectra were collected at a resolution of 1.5 cm^−1^ and an integration time of 500 ms with a single measurement of each point. Collected spectra were normalized and outliers were manually excluded and replaced by averaged surrounding spectra. To differentiate BCP and CPP, collected spectra integrated at crystal-specific peaks at 960 cm^−1^ (BCP) and 1050 cm^−1^ (CPP).

### 2.7 Statistics

Data was analyzed in GraphPad Prism V.9 (GraphPad Prism Software, La Jolla, CA, United States). Normality was determined via Shapiro-Wilk test. Parametric data was compared using a t-test or one-way ANOVA. Non-parametric data was compared by Mann-Whitney or Kruskal–Wallis test. Fisher’s exact test was used to assess gender distribution across radiological calcification grades. Age, Kellgren Lawrence and Krenn Scores were correlated with radiological calcification grade by Spearman correlation. Statistical significance was determined at p < 0.05. Data are presented as median unless stated otherwise.

## 3 Results

### 3.1 Elevated cytokine expression in synovial fluid of patients with radiological calcification

To investigate senescence in respect to articular calcification, we analyzed 68 synovial fluid and 68 synovial membrane samples of a total of 105 OA patients undergoing total knee replacement surgery (Supplementary Figure S2). Patients were grouped by the absence (grade 0) or presence (grades 1–3) of radiological calcification of the knee joint, according to a scoring system we established before (Supplementary Figure S1A) (Stücker et al., 2024).

In this cohort, radiological calcification (grade 1–3) was present in 27% of patients (28/105), while 73% of patients (77/105) showed no signs of calcification (grade 0) on X-rays (Supplementary Figures S1A, B). Radiological calcification was independent of gender (Supplementary Figure S1B). Radiological calcification positively correlated with patients’ age (Supplementary Figure S1C). There was no correlation of radiological calcification grade with Kellgren Lawrence or synovitis measured by Krenn (Supplementary Figures S1D, E).

**FIGURE 1:**
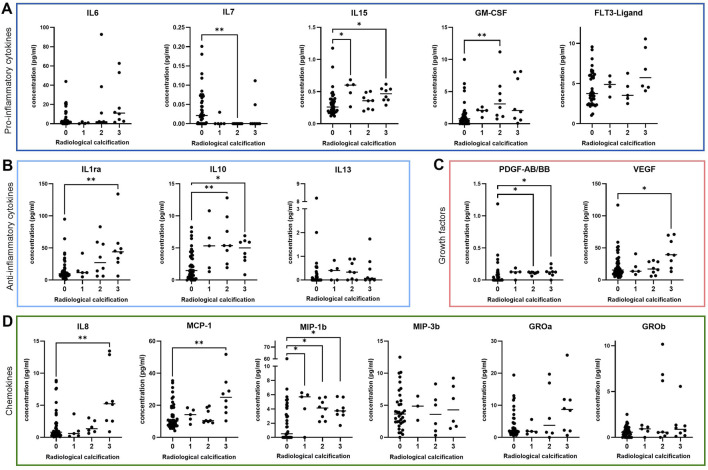
Cytokine expression in synovial fluid of OA patients graded by the severity of radiological calcification. Median concentration of various **(A)** pro-inflammatory cytokines (IL-6: n = 66, IL-7: n = 67, IL-15: n = 64, GM-CSF: n = 67, FLT3-Ligand: n = 55) **(B)** anti-inflammatory cytokines (IL-1ra: n = 66, IL-10: n = 66, IL-13: n = 66) **(C)** growth factors (PDGF-AB/BB: n = 66, VEGF: n = 67) and **(D)** chemokines (IL-8: n = 64, MCP-1: n = 67, MIP-1b: n = 67, MIP-3b: n = 50, GROa: n = 59, GROb: n = 67). * = p ≤ 0.05, ** = p ≤ 0.01.

For analysis of senescence-associated mediators in synovial fluid, we measured the concentration of various cytokines, chemokines and growth factors via magnetic bead-based multiplex assay using Luminex® technology. Synovial fluid concentrations of the pro-inflammatory cytokines IL-15 (p = 0.002) and GM-CSF (p = 0.002) (Figure 1A) and the anti-inflammatory cytokines IL-1 receptor antagonist (IL-1ra) (p = 0.006) and IL-10 (p = 0.001) (Figure 1B) were elevated in patients with evident radiological calcification (grade 2–3). Concentrations of the cytokines IL-6 (p = 0.02), Fms-like tyrosine kinase 3 (FLT3)-Ligand (p = 0.10) and IL-13 (p = 0.12) were slightly elevated in synovial fluid samples from radiologically calcified knee joints, although these trends were not statistically significant (Figures 1A, B). In contrast, synovial fluid levels of IL-7 were downregulated in patients with radiological calcification compared to those without (p = 0.002) (Figure 1A).

In the presence of radiological calcification, synovial fluids also contained elevated levels of the growth factors platelet-derived growth factor AB/BB (PDGF-AB/BB) (p = 0.002) and vascular endothelial growth factor (VEGF) (p = 0.047) (Figure 1C) and the chemokines IL-8 (p = 0.006), monocyte chemoattractant protein-1 (MCP-1) (p = 0.01) and macrophage inflammatory protein-1b (MIP-1b) (p = 0.003) (Figure 1D).

MIP-3b (p = 0.87) and growth-regulated proteins-a (GRO-a) (p = 0.26) and -b (GRO-b) (p = 0.47) levels in synovial fluid did not differ in regards to presence or severity of radiological calcification (Figure 1D).

### 3.2 Similar cytokine profiles in synovial membrane of patients with and without radiological calcification

**FIGURE 2:**
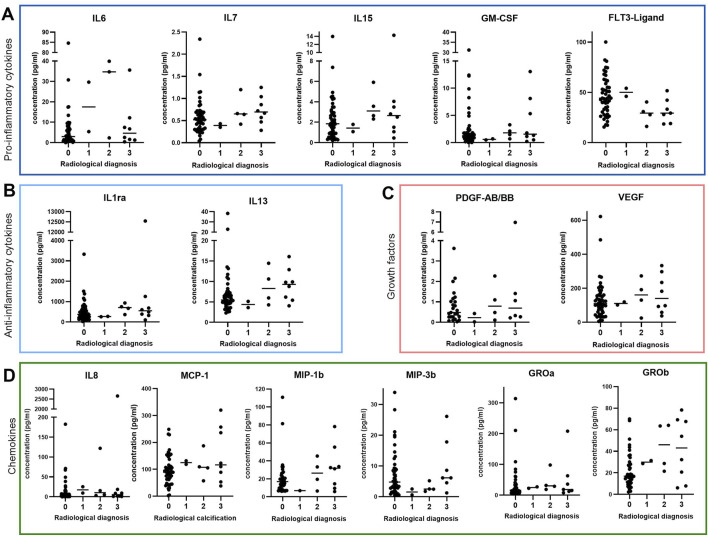
Cytokine expression in synovial membrane of OA patients classified by radiological calcification including various **(A)** pro-inflammatory cytokines (IL-6: n = 64, IL-7: n = 66, IL-15: n = 67, GM-CSF: n = 67, FLT3-Ligand: n = 64), **(B)** anti-inflammatory cytokines (IL-1ra: n = 65, IL-13: n = 65), **(C)** growth factors (PDGF-AB/BB: n = 37, VEGF: n = 64) and **(D)** chemokines (IL-8: n = 66, MCP-1: n = 67, MIP-1b: n = 56, MIP-3b: n = 61, GROa: n = 60, GROb: n = 61).

Since synovial fluid is filtered through the synovial membrane (Tamer, 2013; Ospelt, 2017), we hypothesized that synovial fibroblasts may be the source of the elevated cytokine profile observed in synovial fluid of patients with radiological calcification. Therefore, we measured the above-mentioned cytokines in synovial membrane of OA patients with different radiological calcification grades using the same multiplex assay as before. None of the detected mediators were significantly altered in synovial membrane samples of different radiological calcification grades (Figure 2). Synovial membrane levels of IL-6 (p = 0.17), IL-7 (p = 0.18), IL-13 (p = 0.12) and GRO-b (p = 0.05) were slightly higher in radiologically calcified joints (Figures 2A, B, D), however this was not statistically significant. In contrast, synovial membrane FLT3-ligand levels tended to be higher in the absence of radiological calcification (p = 0.06) (Figure 2A). In contrast to synovial fluid, IL-15 (p = 0.20), GM-CSF (p = 0.34), IL-1ra (p = 0.20), PDGF-AB/BB (p = 0.53), VEGF (p = 0.70), IL-8 (p = 0.24), MCP-1 (p = 0.24) and MIP-1b (p = 0.29) concentrations in synovial membrane were comparable regardless of radiological calcification grade. This was also the case for MIP-3 (p = 0.13) and GRO-a (p = 0.32) (Figure 2D). Synovial membrane levels of IL-10 were below detection limit, so there was no data available.

### 3.3 Elevated synovial fluid cytokine expression is associated with CPP deposition in cartilage

**FIGURE 3:**
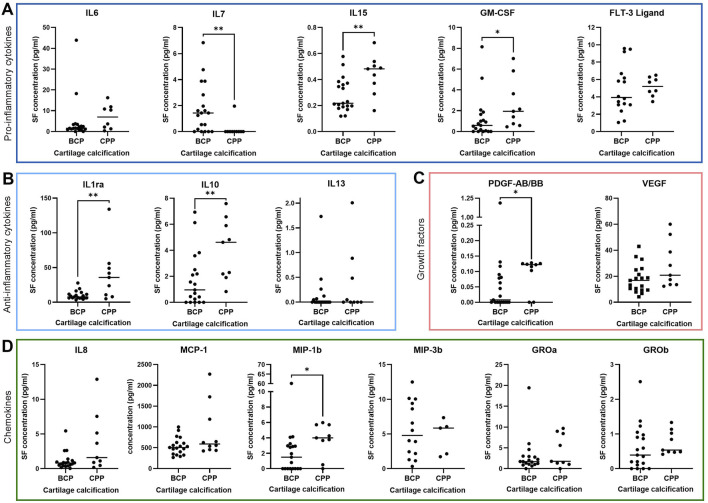
Synovial fluid cytokine expression divided by calcium crystals detected in corresponding calcified cartilage tissue, including various **(A)** pro-inflammatory cytokines (IL-6: n = 27, IL-7: n = 29, IL-15: n = 28, GM-CSF: n = 28, FLT3-Ligand: n = 24) **(B)** anti-inflammatory cytokines (IL-1ra: n = 28, IL-10: n = 28, IL-13: n = 27) **(C)** growth factors (PDGF-AB/BB: n = 28, VEGF: n = 28) and **(D)** chemokines (IL-8: n = 28, MCP-1: n = 29, MIP-1b: n = 28, MIP-3b: n = 19, GROa: n = 27, GROb: n = 28). * = p ≤ 0.05, ** = p ≤ 0.01.

Since we could not detect significant alterations in synovial membrane cytokine profiles in respect to the presence or severity of radiological calcification, elevated concentrations of IL-7, IL-15, GM-CSF, IL-1ra, IL-10, PDGF-AB/BB, VEGF, IL-8, MCP-1 and MIP-1b detected in synovial fluid may originate elsewhere. Thus, we hypothesized cartilage as a potential source of synovial fluid cytokines. Therefore, we determined the crystal type in corresponding calcified cartilage (n = 29). Aiming to establish crystal-specific effects, we differentiated previously measured cytokine concentrations in synovial fluid (Figure 1) according to the type of cartilage calcification. Only 8 of 38 cartilage samples did not contain any calcium crystals on tissue sections despite in some cases displaying calcification on radiographs (Supplementary Figures S2, S3). Therefore, we chose to exclude these samples, eventually comparing synovial fluid cytokine profiles of BCP- and CPP-calcified joints (Figure 3). Interestingly, synovial fluid levels of the majority of the factors that were elevated with radiological calcification (Figure 1) were upregulated in CPP- compared to BCP-calcified joints (Figure 3). Specifically, IL-15 (p = 0.009), GM-CSF (p = 0.04), IL-1ra (p = 0.009), IL-10 (p = 0.01), PDGF-AB/BB (p = 0.04), and MIP-1b (p = 0.02) were more abundant in synovial fluid of patients with CPP compared to BCP calcifications. VEGF (p = 0.06), IL-8 (p = 0.07) and MCP-1 (p = 0.14) showed similar trends towards higher concentrations in the presence of CPP calcifications (Figures 3C, D).

Crystal identification in synovial membrane revealed upregulated levels of IL7, IL15, IL13, IL8 and GROa in the presence of CPP calcification (Supplementary Figure S4).

**FIGURE 4:**
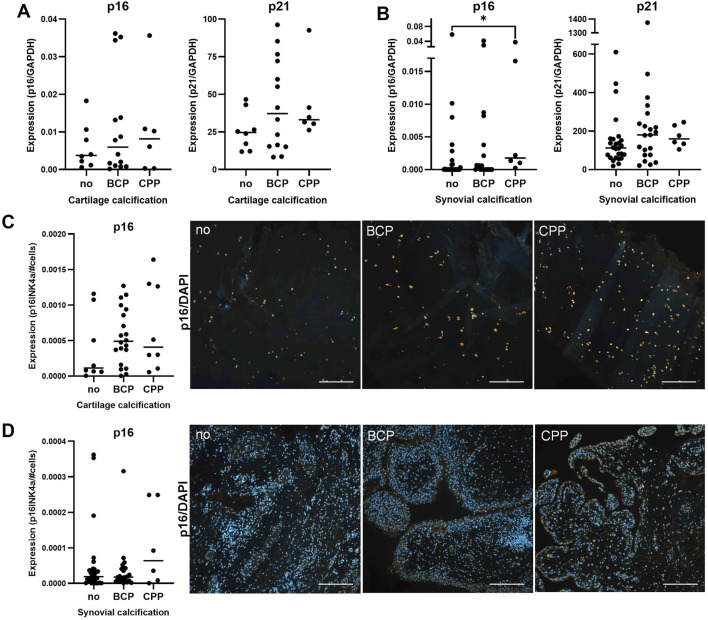
Senescence in non-calcified and calcified cartilage and synovium. **(A)** Median mRNA expression of p16 (p = 0.9, n = 28) and p21 (p = 0.37, n = 28) in OA cartilage. **(B)** Median mRNA expression of p16 (p = 0.04, n = 55) and p21 (p = 0.24, n = 53) in synovium. **(C)** Median of p16-stained area in cartilage (p = 0.39, n = 36) with representative images (right). **(D)** Median of p16-stained area in synovium (p = 0.49, n = 62) with representative images (right).

Articular calcification was also accompanied by cellular senescence, particularly in cartilage tissue. Considering p16 and p21 as markers of senescence, we measured high expression levels at gene and protein level (Figure 4). Both markers were expressed in cartilage and synovial membrane tissue (Figures 4A, B). On protein level, p16 was strongly expressed in chondrocytes and less abundant in synovial cells (Figures 4C, D). Expression levels tended to be upregulated in the presence of calcification, although this trend was not statistically significant.

## 4 Discussion

Local inflammation is a common symptom in joint tissue of OA patients (De Roover et al., 2023). However, inflammatory mediators in the context of articular calcification have rarely been studied. To elucidate how these two processes may be connected, we measured synovial fluid levels of various soluble mediators from knee joints with varying degrees of calcification. In this cohort, radiological calcification was independent of gender and increased with patients’ age. Other studies have demonstrated gender-independent articular calcification before (Hawellek et al., 2016). Regarding the role of age in articular calcification, a review of the literature shows conflicting results. Some studies report calcification to increase with age (Mitsuyama et al., 2007) while others did not show any correlation of these factors (Hawellek et al., 2016).

We found elevated synovial fluid levels of pro-inflammatory cytokines (IL-15, GM-CSF), anti-inflammatory cytokines (IL-1ra, IL-10), growth factors (PDGF-AB/BB, VEGF) and chemokines (IL-8, MCP-1, MIP-1b) in patients with evident radiological calcification of the knee joint. The synovial fluid is filtered through the synovium (Tamer, 2013). However, synovial membrane expression of these inflammatory mediators did not change with radiological calcification, suggesting that these factors may also be produced from cells in the cartilage. Methodologically, radiography lacks both sensitivity and specificity for the detection and localization of intra-articular calcification and cannot differentiate between calcium crystal types (Stücker et al., 2024). Therefore, we applied Raman spectroscopy to identify the type of calcium crystal in calcified joints. This revealed higher levels of various cytokines, chemokines and growth factors in synovial fluid of CPP- compared to BCP-calcified joints, suggesting an association between articular calcification, particularly CPP, and these inflammatory factors.

Chronic inflammation has been suggested as a central player in both OA pathology (Diekman and Loeser, 2024) and calcification (Mas-Bargues et al., 2022). Thus, it is not surprising to find an upregulation of pro-inflammatory cytokines such as IL-15 and GM-CSF in synovial fluid in the presence of articular calcification. In the context of bone mineralization, IL-15 signalling plays a role in osteoblast function and IL-15 receptor knockout impaired bone mineralization *in vivo* and disrupted osteoblast/osteoclast coupling *in vitro* (Loro et al., 2017). IL-15 also stimulated metalloproteinase production in cartilage explants (Warner et al., 2020) and positively correlates with pain and radiographic OA severity (Sun et al., 2013). Interestingly, IL-15 receptor silencing decreased expression and activity of ectonucleotide pyrophosphatase/phosphodiesterase 1 which hydrolyzes ATP into inorganic pyrophosphate (PPi) (Loro et al., 2017). While PPi inhibits BCP calcification, excessive levels promote the formation of CPP crystals (Wuthier et al., 1972). Collectively, these data may suggest a role for IL-15 signaling in articular CPP calcification. In contrast, synovial fluid levels of IL-7 were higher in non-calcified joints. In fact, IL-7 was the only cytokine in our profile that was consistently downregulated in synovial fluid of calcified and particularly in CPP-calcified joints. Interestingly, other studies have shown a decline in IL-7 expression in the blood with increasing age, whereas higher IL-7 levels have been associated with healthy aging and better survival (Passtoors et al., 2015). In view of calcification, IL-7 has been shown to inhibit bone mineral formation *in vitro* and *in vivo* (Weitzmann et al., 2002). Vice versa, blocking of IL-7 was able to prevent postmenopausal bone loss. Thus, downregulated IL-7 levels may enable cartilage calcification via reduced inhibition.

While these results indicate a central role for pro-inflammatory mediators in local ectopic calcification, the role of anti-inflammatory cytokines is less well studied. In this context, we observed upregulated levels of the anti-inflammatory cytokines IL-1ra and IL-10 in synovial fluid in the presence of radiological calcification. As an IL-1 inhibitor, IL-1ra has widely documented inhibitory effects against vascular (Hegner et al., 2018) and articular calcification (Altomare et al., 2021) and has been suggested as a therapeutic agent in these disorders. IL-10 is reportedly expressed in atherosclerotic plaques and exhibits protective effects by reducing cell death and decreasing the development and vulnerability of arterial lesions (Mallat et al., 1999). Single nucleotide polymorphisms in the IL-10 gene have also been associated with valvular calcification (An et al., 2015). IL-10 also effectively downregulates cytokines as IL-6 and IL-8 (Stenvinkel et al., 2005) and both BCP (Grandjean-Laquerriere et al., 2005) and CPP crystals (Campillo-Gimenez et al., 2018) have been shown to trigger its release. Elevated IL-1ra and IL-10 expression in synovial fluid in the presence of radiological calcification may thereby serve to contain the upregulation of the pro-inflammatory factors discussed above.

In addition to cytokines, we also detected elevated concentrations of the growth factors PDGF-AB/BB and VEGF in synovial fluid of CPP-calcified joints. A recent study by Wang and colleagues have associated elevated PDGF-BB levels during aging with cerebrovascular calcification, upregulating osteoblast differentiation genes and the expression of ALP (Wang et al., 2023). Moreover, mutations in PDGF-B can cause calcification in the basal ganglia in mice and humans (Keller et al., 2013). PDGF-BB has further been shown to increase cellular calcium levels and the subsequent production of reactive oxygen species (Lange et al., 2009) that is known to induce senescence. Thereby, PDGF-BB may contribute to both senescence and calcification by disrupting calcium signalling and homeostasis.

Articular calcification was also associated with upregulated synovial fluid levels of chemokines such as IL-8, MCP-1 and MIP-1 that have been implicated in ectopic calcification disorders before. IL-8, for instance promotes aortic valve calcification *in vitro* (Dhayni et al., 2021). In chondrocytes, IL-8 stimulates inorganic phosphate uptake by upregulating the expression of the inorganic phosphate transporter PiT-1 (Cecil et al., 2005). IL-8 production can be induced by GM-CSF and can be synergistically enhanced by CPP crystals (Hachicha et al., 1995). In line with this, GM-CSF has recently been linked to *in vitro* calcification of carotid plaques (Skenteris et al., 2023). MIP-1b has also been associated with vascular calcification, being upregulated in the serum of in patients with coronary artery calcification (Muñoz et al., 2017). *In vitro*, hydroxyapatite nanoparticles, a form of BCP, were able to induce MIP-1b secretion by neutrophils (Velard et al., 2009). Similarly, plasma levels of MCP-1 were upregulated in atherosclerosis patients (Deo et al., 2004) and MCP-1 has been suggested as a marker for coronary artery calcification (Tang et al., 2007).

Beside their involvement in the immune response, most of the above mentioned factors are routinely secreted by senescent cells as part of the characteristic SASP (Coppé et al., 2010; Yue et al., 2022).

Multiple studies have reported increased senescence in cartilage (Gao et al., 2016; Price et al., 2002) of OA patients compared to healthy age-matched individuals. Thus, articular senescence may contribute to the high expression of inflammatory mediators in synovial fluid of calcified OA joints.

Measuring the expression of common senescence biomarkers p16 and p21, we confirmed evident articular senescence, particularly in calcified OA joints. Multiple studies reported a contribution of articular cartilage and chondrocytes to the production of senescence-associated factors. For instance, chondrocytes demonstrated the ability to produce and release reactive oxygen species into synovial fluid, causing oxidative stress, contributing to cartilage degeneration and accelerating senescence (Martin et al., 2004). Chondrocytes are also able to secrete pro-inflammatory cytokines and chemokines such as IL-6 and IL-8 via microvesicles and exosomes (Jenei-Lanzl et al., 2019), supporting cartilage as a potential SASP source. In addition, recent evidence indicates an association between extracellular matrix calcification and chondrocyte senescence via Krüppel-like-factor 10 (Peng et al., 2024). In this context, we have previously shown an upregulation of sortilin and subsequently alkaline phosphatase (ALP) expression in senescent calcifying chondrocytes (Richter et al., 2023).

This study provides evidence for upregulated synovial fluid inflammatory mediators in the presence of articular calcification, particularly CPP deposition. The expression of p16 in OA cartilage and synovial membrane may suggest an association with articular senescence. To test this hypothesis, additional studies are required that analyze the expression of candidate inflammatory factors in cartilage by immunohistochemistry, e.g., showing co-localization with senescence markers or calcification. *In vitro* studies could further measure these inflammatory factors as well as senescence markers in chondrocytes from BCP- and CPP-calcified cartilage to establish a direct link between CPP calcification and senescence (Meyer et al., 2021).

Further, Luminex analysis of cartilage tissue may be interesting to confirm the upregulation of the cytokines that we measured in synovial fluid in the context of CPP calcification. However, articular cartilage has a very low cell density (Stockwell, 1967) compared to synovial tissue (Mehta et al., 2023) and a rather stiff and dense extracellular matrix, probably impeding similar cytokine profiling as performed on synovial fluid and synovial membrane here. In addition, the frequent and extensive cartilage degeneration in advanced OA stages may present an obstacle in terms of sample volume.

Analysis of non-calcified samples would be another interesting addition. A fraction of cartilage samples that we analysed did not show calcification in Raman spectroscopy. However, some of these patients displayed calcification on radiographs, highlighting the spatially restricted view of histological tissue sampling. In this case, Raman measurements were performed on a single tissue of 1 cm diameter and 4 µm thickness. Thus, we cannot exclude the deposition of calcium crystals in the surrounding area, and we are cautious to interpret the data in this group. Taking multiple samples from various locations in the tissue may decrease the risk of missing calcifications and improving representativeness.

Another limitation of this study is the sample size of patients with CPP calcification. As CPP deposition is recognized to be rather rare (Stücker et al., 2024), this limitation is inherent to the crystal type. Collection and analysis of additional samples with CPP calcification would therefore strengthen the presented data.

## 5 Conclusion

Collectively, our data show elevated levels of various inflammatory mediators in synovial fluids of patients with radiological calcification, including many pro- and also some anti-inflammatory cytokines. This upregulation of synovial fluid cytokines may be specifically linked to articular3 CPP-calcification.

## Data Availability

The raw data supporting the conclusions of this article will be made available by the authors, without undue reservation.

## Abbreviations

CPP, Calcium pyrophosphate dihydrate; CPPD, Calcium pyrophosphate deposition disease; BCP, Basic calcium phosphate; SASP, Senescence associated secretory phenotype; SF, Synovial fluid; ALP, alkaline phosphatase; GM-CSF, Granulocyte-macrophage colony-stimulating factor; IL, interleukin; IL-1ra, IL-1 receptor antagonist; MCP-1, monocyte chemoattractant protein-1; MIP-1b/3b, macrophage inflammatory protein-1a/1b; PDGF-AB/BB, platelet-derived growth factor AB/BB; VEGF, vascular endothelial growth factor; CPPD, CPP deposition disease; FLT3-ligand, Fms-like tyrosine kinase 3 ligand; GROa/b, growth-regulated protein a/b.
