# Supplementary figures and images for: CPP-calcification of articular cartilage is associated with elevated cytokine levels in synovial fluid
Source: Front Cell Dev Biol. 2025 Mar 19;13:1535530. doi: 10.3389/fcell.2025.1535530 (PMC11962012; doi:10.3389/fcell.2025.1535530)

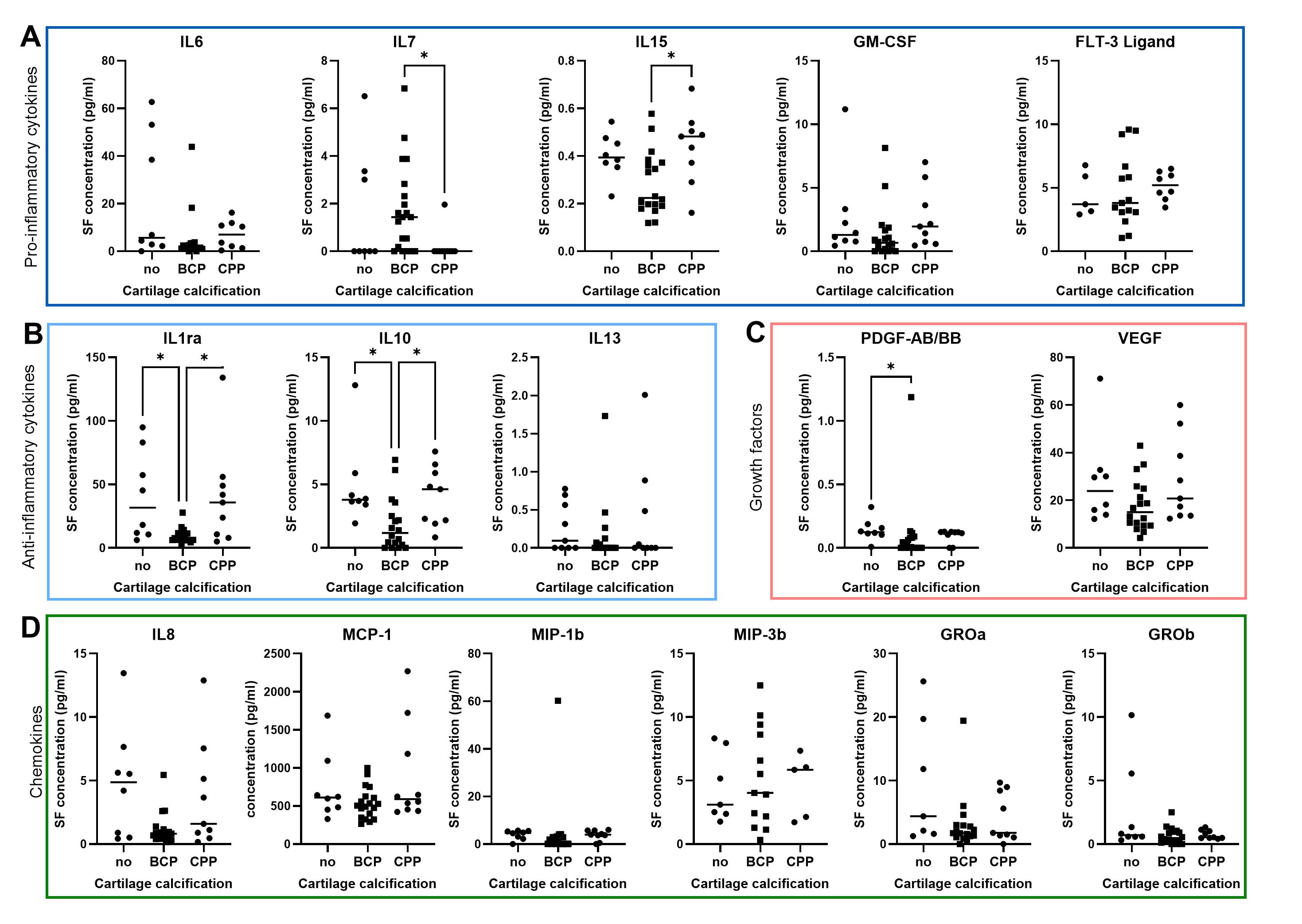

Supplement: Supplementary file 1 [file Image3.jpeg]

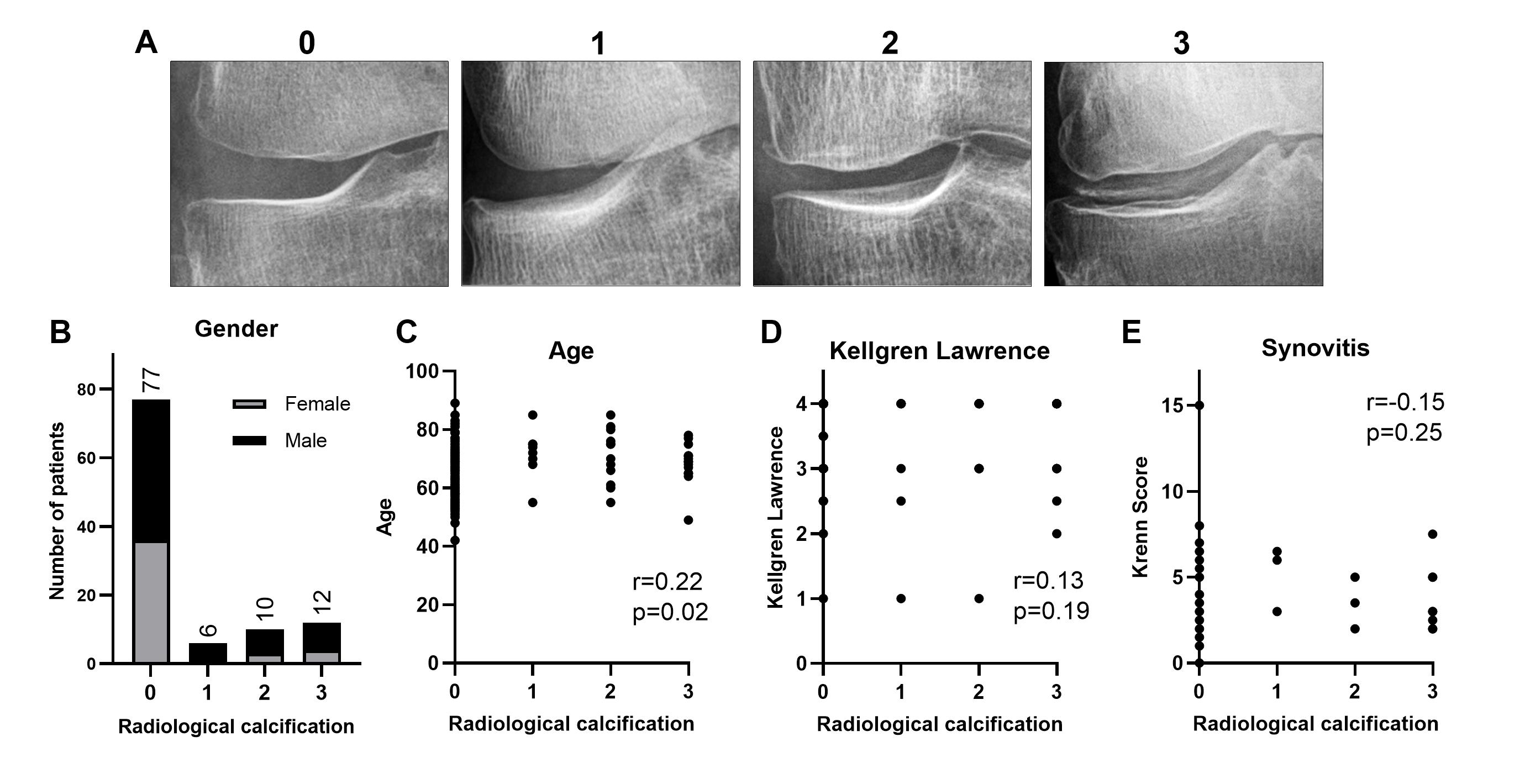

Supplement: Supplementary file 3 [file Image1.jpeg]

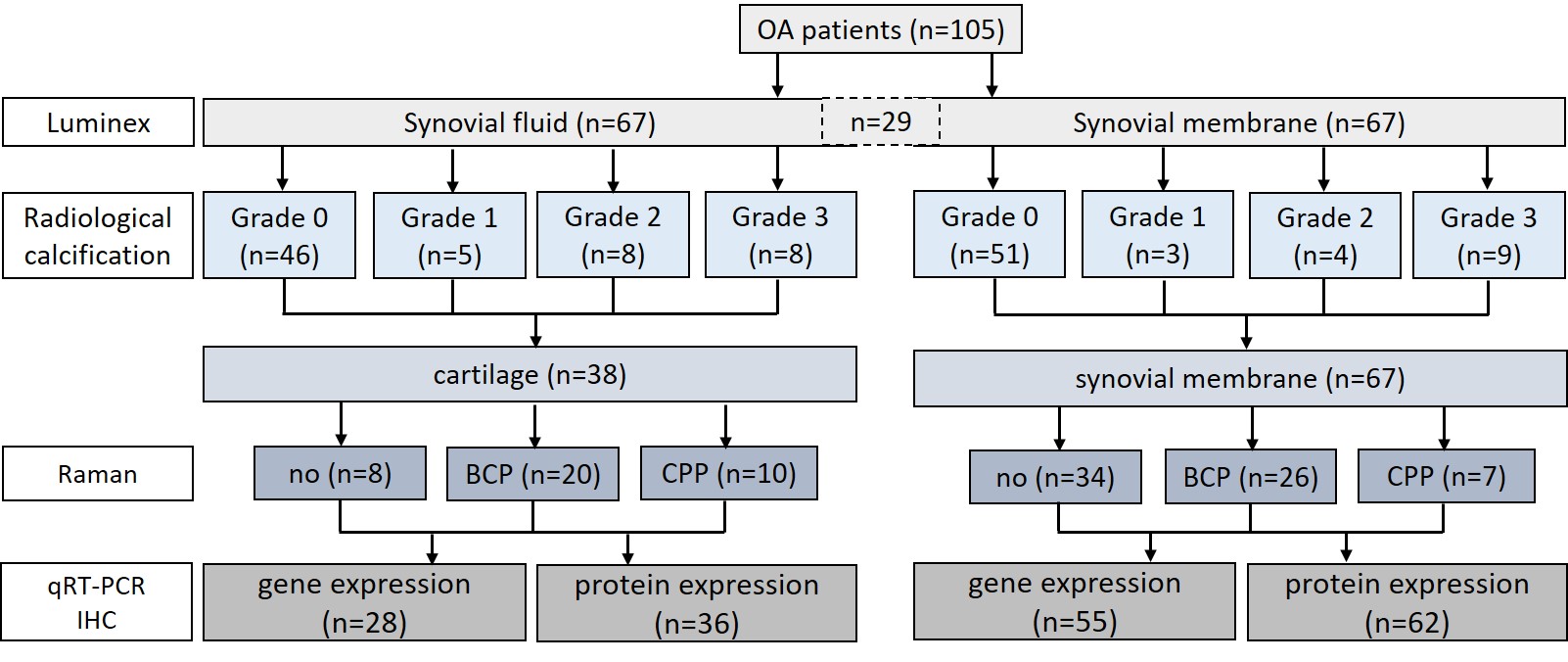

Supplement: Supplementary file 4 [file Image2.jpeg]

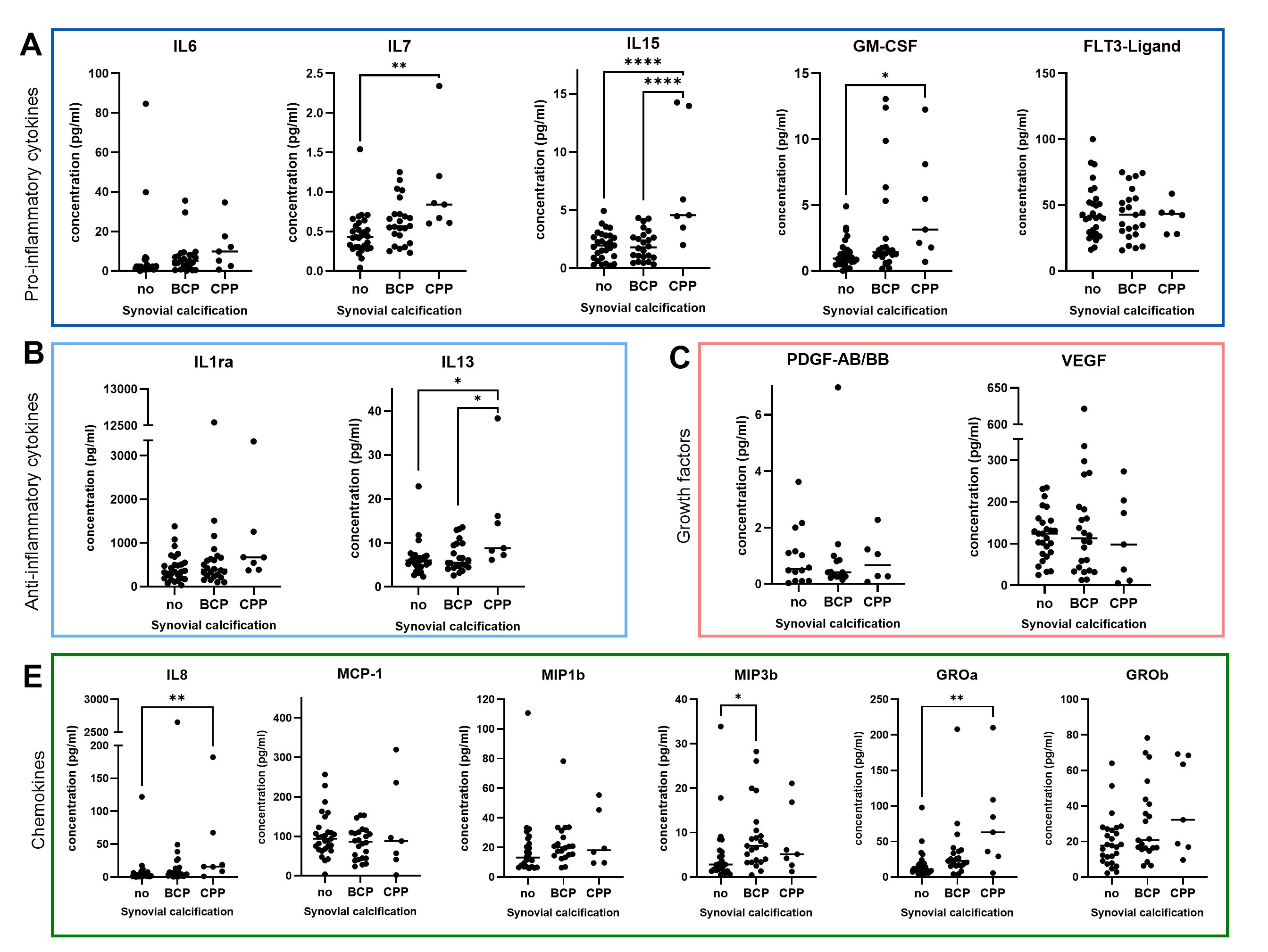

Supplement: Supplementary file 5 [file Image4.png]
