# Supplementary material for: CPP-calcification of articular cartilage is associated with elevated cytokine levels in synovial fluid
Source: Front Cell Dev Biol. 2025 Mar 19;13:1535530. doi: 10.3389/fcell.2025.1535530 (PMC11962012; doi:10.3389/fcell.2025.1535530)
Supplement: Supplementary file 2 [file Table1.docx]

**Supplementary table 1**: Primer sequencese used for qRT-PCR.

| **Gene** | **Product length** | **Sequence (5´-3´)** | |
| --- | --- | --- | --- |
| p16 | 171 | Forward  Reverse | CAA CGC ACC GAA TAG TTA CG  ACC AGC GTG TCC AGG AAG |
| p21 | 108 | Forward  Reverse | GGA GAC TCT CAG GGT CGA AA  CTT CCT GTG GGC GGA TTA |
